# A randomized, controlled field study to assess the efficacy and safety of lotilaner (Credelio™) in controlling fleas in client-owned cats in Europe

**DOI:** 10.1186/s13071-018-2971-9

**Published:** 2018-07-13

**Authors:** Daniela Cavalleri, Martin Murphy, Wolfgang Seewald, Steve Nanchen

**Affiliations:** Elanco Animal Health, Mattenstrasse 24a, 4058 Basel, Switzerland

**Keywords:** Lotilaner, Credelio, Fleas, Fipronil/(S)-methoprene, Frontline, Field, Cat, Efficacy, Safety, Europe

## Abstract

**Background:**

Lotilaner is a new isoxazoline developed as an oral ectoparasiticide for cats and dogs. Its safety, rapid killing onset of action and sustained speed of fleas and ticks kill for a minimum of one month after administration, were demonstrated in a number of laboratory studies in cats.

This study was performed to demonstrate the efficacy and safety of lotilaner flavored chewable tablets for cats (Credelio™, Elanco) in controlling fleas under field conditions in European countries.

**Methods:**

Seventeen veterinary practices in France and Spain, located in high flea prevalence regions, participated in the study. Households with a maximum of three cats and two dogs were randomized 2:1 to a lotilaner (minimum dose rate 6 mg/kg) or a topical fipronil/(S)-methoprene combination (Frontline Combo® Spot-on Cats, Merial) group (administered according to label). In each household, efficacy against fleas and flea allergy dermatitis (FAD) signs were assessed in one primary cat (bearing a minimum of five fleas on Day 0) while safety was evaluated in all cats. There were 121 households included in the lotilaner and 61 in the fipronil/(S)-methoprene groups, respectively. Treatments were administered by the cats’ owners on Day 0. Flea counts and FAD assessments were made on Days 0, 14, and 28. Efficacy calculations were based on geometric mean percent reductions of live flea counts *versus* baseline pre-treatment counts.

**Results:**

Lotilaner efficacy was 97.2 and 98.1% on Days 14 and 28, respectively. Corresponding efficacy for fipronil/(S)-methoprene was 48.3 and 46.4%. Lotilaner was superior to fipronil/(S)-methoprene at all post-Day 0 assessments and over the whole study period (*P* < 0.0001). At every post-administration evaluation, at least 81% of lotilaner-treated cats were flea-free as opposed to 25% in the fipronil/(S)-methoprene group. Lotilaner improved or eliminated clinical signs of FAD, including pruritus. Both products were well tolerated.

**Conclusions:**

Under field conditions in Europe, lotilaner flavored chewable tablets for cats displayed an efficacy against fleas higher than 97%; clinical signs of FAD were improved or eliminated. Lotilaner tablets were safe and provided superior flea control to fipronil/(S)-methoprene.

**Electronic supplementary material:**

The online version of this article (10.1186/s13071-018-2971-9) contains supplementary material, which is available to authorized users.

## Background

The isoxazoline class of compounds are the newest parasiticides marketed for companion animals. These agents differ from other historical parasiticide agents e.g. topically administered compounds, with a new mode of action [1]. Lotilaner, a pure enantiomer of the isoxazoline class, is the newest compound approved for the treatment of flea and tick infestations in dogs (Credelio™ chewable tablets for dogs; Elanco Europe Ltd., Greenfield, IN, USA) [2]. This broad-spectrum parasiticide is a potent inhibitor of the gamma-aminobutyric acid-gated chloride channels, resulting in rapid death of ticks and fleas, after oral administration to dogs [3–5].

Other isoxazolines previously approved for the treatment of flea and tick infestations in dogs since 2014 are afoxolaner, fluralaner and sarolaner. These compounds are available as oral and topical (fluralaner only) formulations. Fluralaner is the first isoxazoline that was approved in cats, formulated as a solution for topical application (Bravecto® spot-on solution for cats; Merck Animal Health, Madison, NJ, USA) [6]. Currently there is no isoxazoline-containing ectoparasiticide product for oral administration available for the treatment of fleas and tick infestations in cats.

During a market research conducted as part of the development of lotilaner for cats (unpublished data), pet owners expressed specific, negative emotions related to the administration of topical spot-on products to cats and the disruption that occurs in the bond between the owner and their cat when topical products are applied. Many of these owners responded positively to the idea of an easy-to-give flavoured, oral tick and flea option for cats. A small, flavoured, cat-friendly, oral tablet would, therefore, be a welcomed novel product filling the gap in tick and flea control in cats.

In a number of pivotal laboratory studies, the safety and the efficacy of lotilaner flavored chewable tablets for cats (Credelio™, Elanco) against fleas (*C. felis*) and ticks (*Ixodes ricinus*) for 1 month, following oral administration at the minimum dose rate of 6.0 mg/kg, was demonstrated [7, 8].

A pivotal tolerance study in 8-week-old kittens had shown lotilaner tablets to be safe at doses up to 130 mg lotilaner/kg (high dose of 130 mg/kg; actual high dose levels of 131.24 mg/kg for males and 131.30 mg/kg, respectively for females) for monthly treatment over 8 months [9].

In this study, the authors evaluated the efficacy and safety of lotilaner administered once, at the dose rates intended for the marketed product (6.0 to 22.9 mg/kg body weight), to cats naturally infested with fleas under field conditions in Europe. A fipronil/(S)-methoprene combination (Frontline Combo® Spot-on Cats, Merial, Lyon, France) was used as the positive control. The effect of the product on clinical signs associated with flea allergy dermatitis (FAD) was also evaluated.

## Methods

This assessor-blinded, randomized, positive-controlled, non-inferiority, multicentre field trial was conducted according to the study authorizations issued by the Agencia Española de Medicamentos y Productos Sanitarios (Spanish regulatory authorities) and the Agence Française de Sécurité Sanitaire des aliments (AFSSA) (French regulatory authorities), and in compliance with the applicable regulatory guidelines, which were current at the time the study was performed [10–15].

### Animals

Seventeen veterinary practices in France and Spain participated in the study. Sites were selected in areas with a known high prevalence of fleas. Households with a maximum of three cats and two dogs were eligible to participate, provided that cats and dogs did not regularly or frequently contact each other or share resting places, for the whole duration of the study.

Cats aged ≥ 8 weeks and weighing ≥ 1 kg were eligible for enrolment. At least one cat from each household (primary cat) had to be found to be infested with ≥ 5 fleas prior to treatment. All cats were required to be clinically healthy or with conditions judged not to interfere with the study by the study veterinarian. Inclusion of cats showing signs of FAD was encouraged.

Cats with known hypersensitivity to the active ingredients and/or excipients of the investigational veterinary product: Credelio™ (lotilaner chewable tablets for cats, Elanco, Greenfield, IN, USA) or control product (Frontline Combo® Spot-on Cat, Merial, Duluth, Georgia) were not eligible for inclusion in the study. Pre-treatments with other ectoparasiticide compounds, pregnancy or lactation were criteria that further excluded cats, as well as planned routine surgical procedures, until cats fully recovered from any intervention and no influence on the study procedures was expected. Other exclusion criteria were plans for the animals to be used for breeding within 4 months of treatment, convalescence from any serious conditions, pre-existing medical and/or surgical conditions other than flea infestation and FAD (unless such conditions did not interfere with the suitability for the study treatments administration, were mild or chronic, stable and under control, according to the judgment of the examining veterinarian). During the study, animals could be withdrawn due to concomitant disease, death or euthanasia, or serious adverse events (SAEs) not compatible with the study. Early withdrawal could also result from non-compliance with the protocol, owner decision, or pre-termination of the study as decided by the sponsor.

All animals stayed with their owners throughout the study. The participating households were not permitted to use any environmental treatments to control flea infestations during this period. All animals were provided with food and water per the owners’ usual practices.

### Randomisation and treatment

At each site, cats were randomised per household in the sequence of inclusion according to the random treatment allocation plan. All cats from the same household were randomized to the same treatment. The random treatment allocation plan was created using a block design and a 2:1 ratio (lotilaner:fipronil/(S)-methoprene). The target number of enrolled subjects for efficacy analysis (primary cats) was 180, divided 2:1 between lotilaner-treated subjects and fipronil/(S)-methoprene-treated subjects. In each household, there could be one primary cat only; any other cats (up to two) in the same household were supplementary cats, treated with the same product as the primary cat but only assessed for safety.

Treatment was administered once, on Day 0 of the study by the animals’ owners. All animals in Group 1 received Credelio™ and all animals in Group 2 received Frontline Combo® Spot-on Cat. Credelio™ was administered orally within 30 min following feeding. The tablets (strengths: 12 or 48 mg lotilaner) were administered based on each cat’s individual body weight to achieve a minimum dose rate of 6.0 mg/kg and a maximum of 22.9 mg/kg. Frontline Combo® Spot-on Cat (fipronil 50 mg/(S)-methoprene 60 mg) was administered topically per the manufacturer’s product label, applied as a single 0.5 ml pipette regardless of body weight. Dogs (maximum of two per household) and other animals in the household posing a risk of flea transmission to cats were to be treated with a suitable oral ectoparasiticide efficacious against fleas.

### Study assessments

This study evaluated the efficacy against fleas and safety of lotilaner chewable tablets compared with Frontline Combo® Spot-on Cats, both administered once, to cats naturally infested with fleas. The effect of the product on clinical signs associated with FAD was also evaluated. All efficacy analyses were performed for the primary cats whereas safety analyses were performed for all cats enrolled in the study.

The primary efficacy criterion was the average efficacy of lotilaner compared with fipronil/(S)-methoprene over the entire treatment period, as based on flea counts for each visit compared with baseline flea counts, averaged on all visits, in a non-inferiority test. The secondary efficacy criteria were efficacy of the lotilaner compared with the control product for each visit, based again on the comparison between post-treatment and baseline flea counts, and the assessment of FAD signs for primary cats with FAD on Day 0. All efficacy analyses were performed on 14 (± 2) and 28 (± 2) days post-treatment.

A full body flea count was performed for each cat with a flea comb per the procedure defined in the protocol. Each cat was combed for at least 10 min, and combing continued for another 5 min after the last flea was found. In the event that more than 100 fleas were counted and the counting was not finished, the total number of fleas was recorded as > 100. All cats (primary and secondary) were assessed for safety based on health observations for 28 (± 2) days post-treatment. In addition, primary cats with clinical signs of FAD were assessed for signs of FAD on Days 0, 14 (± 2), and 28 (± 2). FAD signs (alopecia, crusts, erythema, hyperpigmentation, miliary dermatitis, eosinophilic granuloma, eosinophilic plaque, eosinophilic ulcer, papules, pruritus and scales) were classified as absent, mild, moderate, or severe and assigned a score from 0 (absent) to 3 (severe) by the Investigator. For the sign “pruritus”, the scoring was done as follows: absent, no scratching; mild, occasionally scratching; moderate, frequently scratching and/or biting itself; and severe, intense scratching/biting itself. Animals were observed for AEs (adverse events) for the whole duration of the study.

The environmental pressure of flea infestation at the sites where the trial was conducted was also evaluated throughout the study, based on the estimated overall number of animals (cats and dogs) presented in the veterinary practice or clinic diagnosed with a flea infestation as well as the estimated number of products supplied for flea prophylaxis and/or treatment in the last 7 days prior to the study visit of a cat.

### Statistical analyses

All study animals were divided into the following three analysis sets: intent-to-treat (ITT) efficacy population, comprising all subjects that were randomized to a treatment and that presented with ≥ 5 fleas at inclusion (one cat per household, primary cat); per protocol (PP) efficacy population, comprising subjects (primary cat) without major protocol deviations; safety population, comprising all subjects that were randomized to a treatment and received one dose of lotilaner or the fipronil/(S)-methoprene (primary and supplementary cats).

The Clinsight® Electronic Data Capture System was used for data collection. All calculations were performed using SAS® version 9.2 (SAS Institute Inc., Cary, NC, USA). The statistical hypotheses were tested on a 2-sided level of significance of 0.05. *P*-values ≤ 0.05 were considered significant.

For the demographics and related variables such as sex, age, body weight, breed, hair length, and the time animal spends indoor/outdoor, summary statistics and/or frequencies were calculated and the two groups were compared with a non-parametric test (Kruskal-Wallis, Mann-Whitney, or Fisher’s exact test, depending on the parameter).

Efficacy endpoints were assessed in the two efficacy populations (ITT and PP). Percent efficacy was defined in relation to baseline values, i.e.

$$ \%\mathrm{Efficacy}=100\times \left(\mathrm{Flea}\ \mathrm{count}\ \mathrm{day}\ 0-\mathrm{Flea}\ \mathrm{count}\ \mathrm{actual}\ \mathrm{day}\right)/\left(\mathrm{Flea}\ \mathrm{count}\ \mathrm{day}\ 0\right) $$

Flea counts recorded as “higher than 100” were assigned a nominative value of 101 for the purposes of statistical analysis. Flea counts and reduction of flea counts *versus* baseline were analysed statistically. Summary statistics including arithmetic and geometric mean, minimum, maximum, and median were provided for all parameters of interest. Treatment groups were compared by analysis of covariance (ANCOVA) methods, on original scale or after possible log-transformation. To avoid taking the log of zero, one (1) was added to all flea counts before log-transformation. In the ANCOVA, the number of cats per household was used as a covariate. Non-inferiority was claimed when the 2-sided 95% confidence interval (CI) for the ratio of flea counts for lotilaner, divided by the same value for Frontline Combo® Spot-on Cats, was within the interval [0, 1/0.80] or [0, 1.25]. This indicated that the results showed (with 97.5% confidence) that flea counts with lotilaner were not higher than flea counts with Frontline Combo® Spot-on Cats, up to a non-inferiority margin of 20%.

Safety endpoints were assessed in the safety population on Days 0, 14 (± 2 days; primary cats only) and Day 28 (± 2 days; all animals). The cats were observed for AEs, SAEs, and changes in body weight. Summary statistics including arithmetic and geometric means, minimum, maximum, and median were calculated for all parameters of interest. Treatment groups were compared by analysis of variance (ANOVA) methods; body weight data were log-transformed in order to improve normality. Adverse events were counted in each group and classified using the VeDDRA coding system. The relationship with the product administration was assessed according to the ABON classification (A, probable; B, possible; O, unclassified/unknown; N, unlikely/unrelated) both by the examining veterinarian and the sponsor representative.

French translation of the Abstract is available in Additional file 1.

## Results

### Animals

A total of 320 cats (182 primary and 138 secondary), from 182 households, were randomised to either treatment at 17 veterinary practices in France and Spain. The majority of primary cats (*n* = 83; 46%) belonged to households where only one cat was included in the study, followed by households with two cats enrolled (*n* = 60; 33%), and by households with three cats (*n* = 39; 21%).

Efficacy evaluation was performed on primary cats only, in the ITT and PP populations. The ITT population comprised all primary cats included in the study (*n* = 182; 121cats in the lotilaner group and 61 in the control group). The PP population comprised 178 primary cats (120 and 58 in the lotilaner group and control group, respectively) as four animals had deviations that prevented their inclusion in the PP analysis. One cat was excluded for one visit only (Day 14). All 320 cats (primary and secondary) were analysed for safety, comprising 217 cats in the lotilaner group and 103 cats in the control group.

The efficacy results obtained in primary cats were almost identical for the ITT population (*n* = 182 cats) and the PP population (*n* = 178 cats); therefore, only efficacy results of the ITT population are presented here.

Both treatment groups from the ITT population were homogeneous for all variables analysed prior to treatment administration: sex (*Z* = 0.254, *P* = 0.8741); age (*Z* = 0.452, *P* = 0.6510); body weight (*Z* = 0.267, *P* = 0.7896); breed (*χ*^2^ = 12.30, *df* = 7, *P* = 0.0911); hair length (*Z* = 0.991, *P* = 0.3216); lifestyle (mostly indoors, mostly outdoors, indoors and outdoors; *χ*^2^ = 2.66, *df* = 2, *P* = 0.2650); number of cats in the household (*Z* = 0.900, *P* = 0.3680); and flea counts (*t*_(178)_ = 0.50, *P* = 0.6159) (Table 1). Results for the safety population were similar, except for the breed variable (*χ*^2^ = 15.34, *df* = 7, *P* = 0.0319), with more European cats in the lotilaner group (23%) compared with the fipronil/(S)-methoprene group (13%). Seven different pure breeds of cats were included in the ITT population, of which the most common were European (*n* = 38; 21%), Persian (*n* = 6; 3%), and Siamese (*n* = 4; 2%). All cats enrolled in the study were successfully dosed by their owners.

**Table 1:** Demographics and baseline characteristics of the enrolled animals (ITT population)

		Lotilaner-treated animals (*n* = 121)	Fipronil/(S)-methoprene-treated animals (*n* = 61)	Comparison	
				*Z*-value^a^/ *χ*^2^-value^a^/ *t*-value	*P*-value
Age (years)	Mean ± SD	3.49 ± 3.12	3.42 ± 3.57	*Z* = 0.452	0.6510
	Range	0.17–13.0	0.17–14.0		
Weight (kg)	Mean ± SD	3.8 ± 1.5	3.9 ± 1.7	*Z* = 0.267	0.7896
	Range	1.0–8.7	1.1–8.0		
Sex	Male	69 (57%)	36 (59%)	*Z* = 0.254	0.8741
	Female	52 (43%)	25 (41%)		
Breed	European	29 (24%)	9 (15%)	*χ*^2^ = 12.30, *df* = 7	0.0911
	Persian	4 (3%)	2 (3%)		
	Siamese	0 (0%)	4 (7%)		
	Maine Coon	2 (2%)	0 (0%)		
	Ragdoll	1 (1%)	0 (0%)		
	Chartreuse	1 (1%)	0 (0%)		
	Selkirk rex	1 (1%)	0 (0%)		
	Crossbreeds	83 (69%)	46 (75%)		
Animal spends time	Mostly indoors	48 (40%)	19 (31%)	*χ*^2^ = 2.66, *df* = 2	0.2650
	Indoors and outdoors	56 (46%)	36 (59%)		
	Mostly outdoors	17 (14%)	6 (10%)		
No. of cats in household	1	52 (43%)	31 (51%)	*Z* = 0.900	0.3680
	2	42 (35%)	18 (30%)		
	3	27 (22%)	12 (20%)		
	Mean ± SD	1.8 ± 0.8	1.7 ± 0.8		
	Range	1.0–3.0	1.0–3.0		
Hair length	Short	76 (63%)	42 (69%)	*Z* = 0.991	0.3216
	Medium	27 (22%)	14 (23%)		
	Long	18 (15%)	5 (8%)		
Day 0 flea count	Mean ± SD	15.75 ± 17.54	16.92 ± 18.22	*t*_(178)_ = 0.50	0.6159

^a^*Z* of a Mann-Whitney test; *χ*^2^ of a Kruskal-Wallis test (of treatment *vs* breed); *df*, degrees of freedom; *t*_(*df*)_, ANOVA t-statistic with *df* degrees of freedom

*Abbreviation*: *SD* standard deviation

One cat from each of the treatment groups was prematurely withdrawn from the study: in the lotilaner-treated group, a supplementary cat died on Day 23 after being run over by a car, a primary cat from the fipronil/(S)-methoprene-treated group died on Day 3 following presentation with clinical signs of dehydration and severe dyspnoea.

### Flea efficacy assessment

**Fig. 1:**
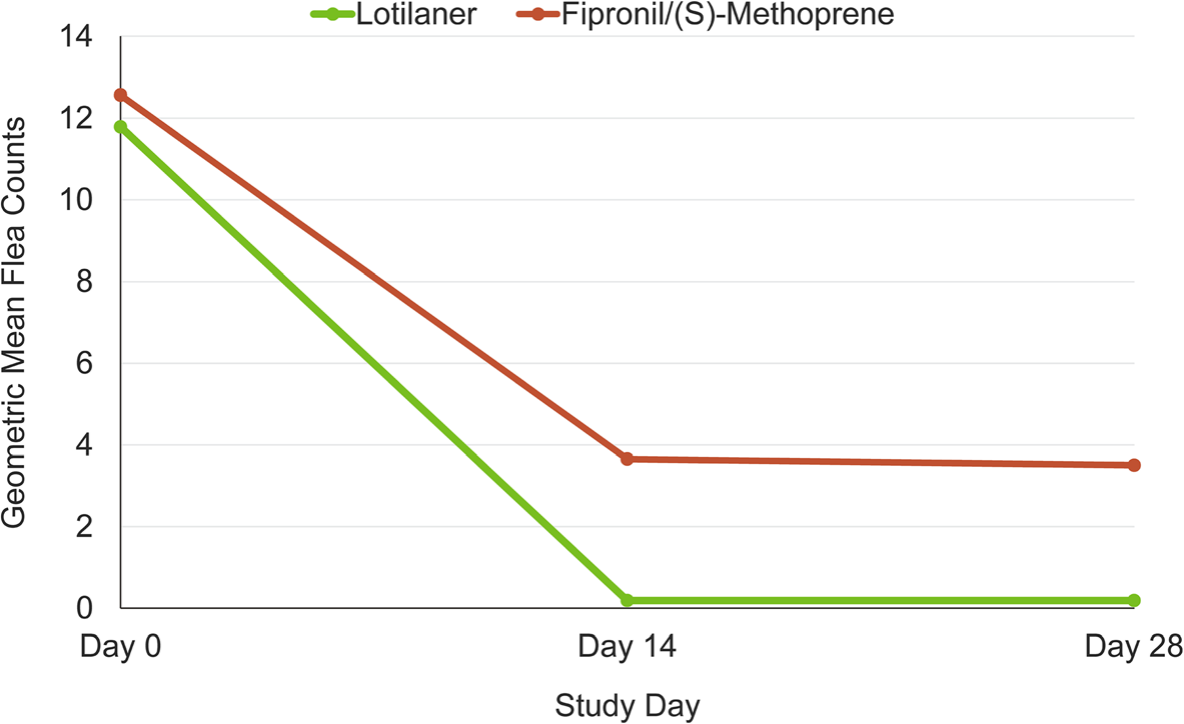
Geometric mean flea counts of lotilaner- and fipronil/(S)-methoprene-treated cats at each assessment time-point. Difference between groups was significant: *t*_(176)_≥ 11.5, *P* < 0.0001

The average arithmetic (± standard deviation, SD) and geometric mean flea counts over the study period were, respectively, 0.41 and 0.19 in the lotilaner-treated group and 8.87 and 3.59 in the fipronil/(S)-methoprene-treated group. The arithmetic and geometric mean flea counts over time are displayed in Table 2. The geometric mean flea counts over time are also shown in Fig. 1.

**Table 2:** Flea count data for each treatment group

	Flea count	Lotilaner	Fipronil/(S)-methoprene	95% CI^a^
Day 0	Arithmetic mean ± SD	15.75 ± 17.54	16.92 ± 18.22	na
	Range	5–101	5–101	
	Geometric mean	11.79	12.56	
Day 14	Arithmetic mean ± SD	0.49 ± 2.69	8.35 ± 15.21	0.21–0.33
	Range	0–29	0–101	
	Geometric mean	0.19	3.66	
Day 28	Arithmetic mean ± SD	0.33 ± 1.02	9.38 ± 19.07	0.21–0.34
	Range	0–8	0–101	
	Geometric mean	0.19	3.51	

^a^Calculated as lotilaner/ fipronil/(S)-methoprene

*Abbreviations*: *CI* confidence interval, *SD* standard deviation, *na* not applicable

**Fig. 2:**
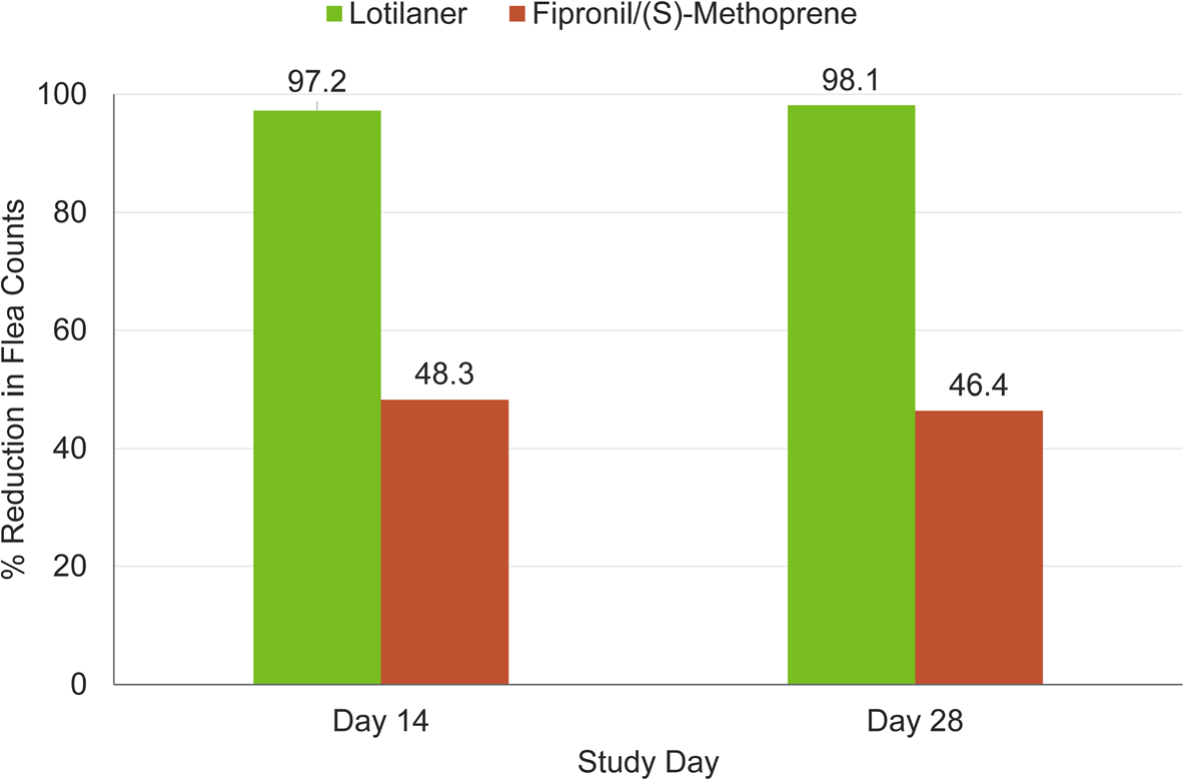
Geometric mean percent flea reduction of lotilaner- and fipronil/(S)-methoprene-treated cats at each assessment time-point. Difference between groups was significant: *P* < 0.0001 (*t*_(176)_ = 7.96 and *t*_(176)_ = 8.13 on days 14 and 28, respectively)

The overall geometric mean percentage flea reduction for the study period was 97.7% in cats treated with lotilaner compared with a reduction of 47.4% for cats treated with fipronil/(S)-methoprene. Percentage flea reductions for each assessment time-point are presented in Fig. 2.

ANCOVA analysis of post-treatment flea counts and percentage reductions in flea counts, including 95% confidence intervals (CIs), showed significant reductions in the lotilaner-treated cats on Days 14 and 28 and over the whole study (*P* < 0.0001) compared with the control product-treated animals. Analysis of the CIs for flea counts revealed that not only non-inferiority to fipronil/(S)-methoprene could be shown for lotilaner (i.e. upper confidence limit was below 1.25); superiority could be demonstrated at all time-points and for the entire study period as well (*P* < 0.0001).

In the lotilaner group, 81.0 and 81.8% cats were flea-free on Day 14 and 28, respectively. In the fipronil/(S)-methoprene group, 25.0% cats were flea-free at the same time-points (Table 3).

**Table 3:** Number and percentage of flea-free (cured) cats at each time-point

Day	Group 1: lotilaner			Group 2: fipronil/(S)-methoprene		
	*n*	Cured	Percent	*n*	Cured	Percent
14	121	98	81.0	60	15	25.0
28	121	99	81.8	60	15	25.0

### FAD assessment

Assessment of FAD signs for primary cats with FAD on Day 0 was performed on ten cats in the lotilaner-treated group and six cats in the fipronil/(S)-methoprene-treated group. Baseline analysis of clinical signs of FAD prior to start of treatment administration did not reveal any statistically significant differences between treatment groups, thus confirming that they were balanced at the beginning of the study. All clinical signs associated with FAD could be evaluated during the study except eosinophilic granuloma, which was not observed in any of the study animals evaluated for FAD.

**Fig. 3:**
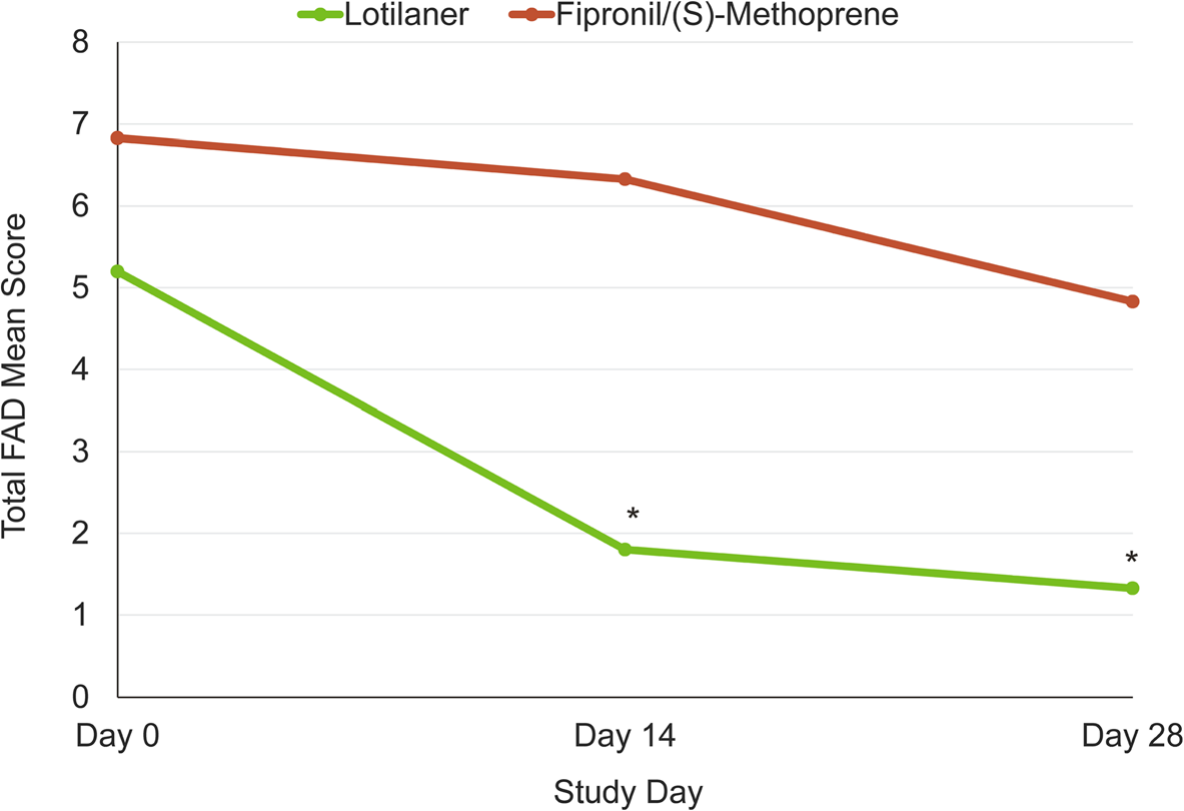
FAD mean scores of lotilaner- and fipronil/(S)-methoprene-treated cats at each assessment time-point. Statistically significant difference from baseline: *S ≥ 22.5, *P* ≤ 0.0039

In the lotilaner group, there was a significant decrease in the mean total FAD score on Day 14 and 28 (Wilcoxon paired-sample test: *S* = 22.5, *P* = 0.0039 for day 14; *S* = 27.5, *P* = 0.0020 for day 28); on Day 0 the score was 5.2, which declined to 1.8 by Day 14 and 1.3 at the end of the study. In the fipronil/(S)-methoprene group, the mean total FAD score decreased from 6.8 on Day 0, to 6.3 and 4.8 on Days 14 and 28 respectively, and appeared to be not statistically significant (*S* = 4.5 and 6.5, *P* = 0.41 and 0.25 on Days 14 and 28, respectively) but due to the low number of animals, statistical significance could not be definitively assessed (Fig. 3).

**Fig. 4:**
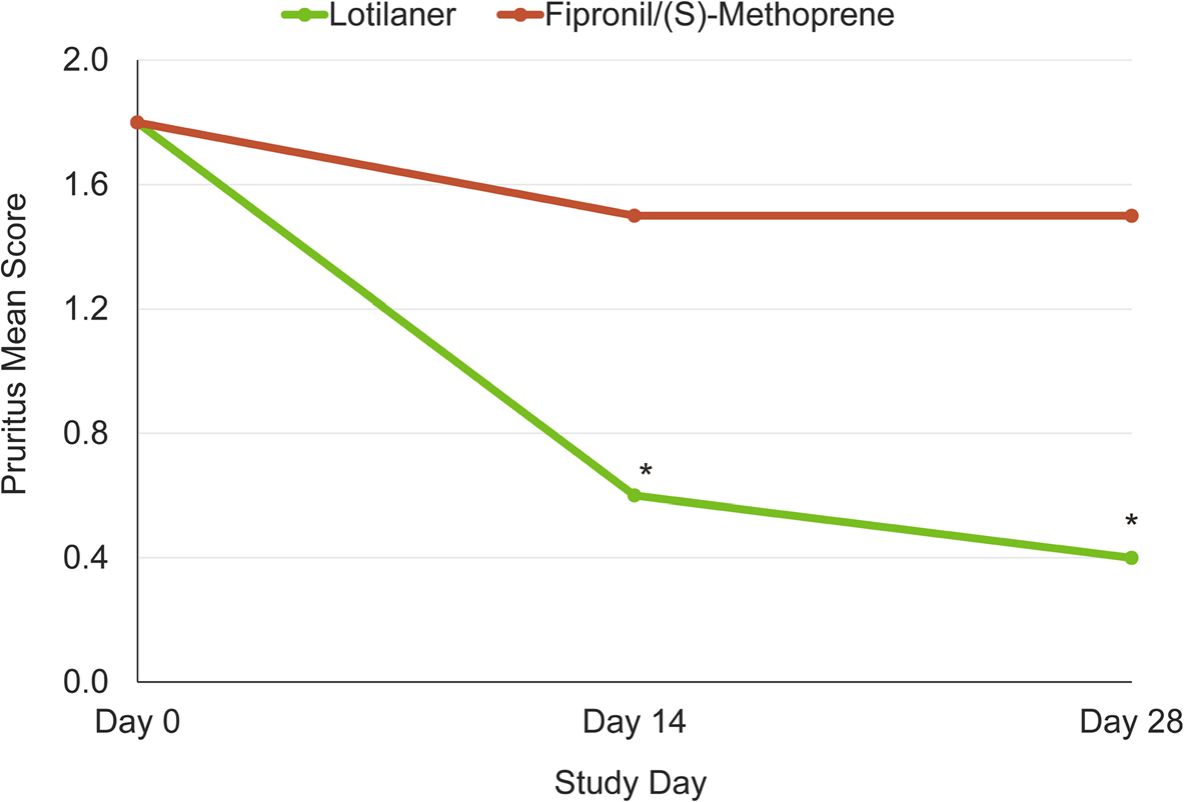
Pruritus mean scores of lotilaner- and fipronil/(S)-methoprene-treated cats at each assessment time-point. Statistically significant difference from baseline: *S ≥ 22.5, *P* ≤ 0.0039

Pruritus mean scores followed the same pattern as mean total FAD scores, decreasing significantly, in the lotilaner group, from 1.8 on Day 0 to 0.6 and 0.4 on Days 14 and 28, respectively (*S* = 22.5, *P* = 0.0039 for day 14; *S* = 27.5, *P* = 0.0020 for day 28). In the fipronil/(S)-methoprene group the decrease from 1.8 (Day 0) to 1.5 (Day 14 and Day 28) was not significant (*S* = 2.5 and 1.5, *P* = 0.6250 and 0.7500, respectively) (Fig. 4). Statistically significant differences were also observed between the lotilaner and control groups, in the pruritus score (*t*_(12)_ = 2.50 and 3.71, *P* = 0.0281 and *P* = 0.00340 on Days 14 and 28, respectively), and in the total FAD score (*t*_(12)_ = 3.11, *P* = 0.0091), averaged over the entire study duration, with lower scores in the lotilaner group.

### Safety

Safety was evaluated in 320 cats (182 primary and 138 secondary) enrolled in the study and included 217 cats that were treated with lotilaner and 103 cats treated with fipronil/(S)-methoprene.

Fifteen out of the 217 cats in the lotilaner-treated group (6.91%) and five of 103 cats (4.85%) in the fipronil/(S)-methoprene-treated group were affected by non-serious, mild adverse events.

Four animals had SAEs (three in the lotilaner group and one in the control group: 0.014% and 0.010%, respectively) during the study. Signs included abdominal pain, digestive tract stenosis and obstruction, urinary tract obstruction, dyspnoea, pyothorax, dehydration, lethargy and death. Two cats died during the study - one cat in the lotilaner group was run over by a car and one cat in the fipronil/(S)-methoprene group was diagnosed with pyothorax. Two other cats in the lotilaner group presented one with urinary tract obstruction requiring surgical intervention and the other one with the presence of a foreign body in the gastrointestinal tract requiring surgery. These cats made a full recovery post-intervention and completed the study. None of the SAEs was assessed as being related to the study treatment.

Fisher’s exact test showed that the number of cats affected by adverse events or serious adverse events was not significantly different between the two groups for each of the signs (*Z* ≤ 2.05, *P* ≥ 0.1029 and *Z* ≤ 1.44, *P* ≥ 0.3219, respectively).

The average body weight of the lotilaner-treated cats was 3.95 kg (SD 1.59, range 1.00–10.50 kg) and for the fipronil/(S)-methoprene-treated cats was 3.89 kg (SD 1.57, range 1.00–8.00 kg), at baseline (Day 0). There were no significant differences between treatment groups in body weights of cats on Day 0 (*t*_(160)_ = 0.12, *P* = 0.9064) and body weights as well as body weight gain on Days 14 and 28 (*t*_(176)_ = 1.76, *P* = 0.0798 and 0.8177, for Day 14; *t*_(154)_ = 0.23, *P* = 0.8177, for Day 28); see Table 4.

**Table 4:** Mean body weight and body weight changes over time

	Day	Credelio™		Frontline Combo® Spot-on Cat	
		*n*	Mean ± SD	*n*	Mean ± SD
Body weight (kg)	0	217	3.95 ± 1.59	103	3.89 ± 1.57
	14^a^	121	3.84 ± 1.40	60	3.99 ± 1.61
	28^b^	216	4.08 ± 1.47	102	4.02 ± 1.43
Body weight gain (kg)	14^a^	121	0.06 ± 0.18	60	0.10 ± 0.19
	28^b^	216	0.11 ± 0.25	102	0.13 ± 0.24

^a^Primary dogs only

^b^Primary and supplementary dogs

### Environmental pressure

Data on environmental pressure in the week before a scheduled visit were recorded on Days 0, 14 (± 2) and 28 (± 2). The number of animals (cats and dogs) diagnosed with a flea infestation during the last 7 days prior to each case visit ranged over all study sites between eight (week of 26 October 2015) and 32 cases (week of 20 July 2015). The estimated average number of products supplied at the clinic for flea prophylaxis and/or treatment in the last 7 days prior to the study visit of a cat ranged between 21 (week of 26 October 2015) and 97 (week of 20 July 2015), while the estimated average number of animals (cats and dogs) diagnosed with a flea infestation, ranged, over all study sites and countries between 8 (week of 26 October 2015) and 32 cases (week of 20 July 2015).

## Discussion

Both lotilaner and fipronil/(S)-methoprene groups demonstrated post-treatment flea counts reduction. Results showed that cats treated with lotilaner had significantly lower flea counts on Days 14 and 28 and over the entire study (*P* < 0.0001) compared with animals treated with fipronil/(S)-methoprene. Credelio™ was shown to be superior to Frontline Combo® Spot-on (*P* < 0.0001) at both time points and on average.

A percentage of 6.91 Credelio™ cats and 4.85 cats treated with Frontline Combo® Spot-on were affected by adverse events. The difference was not statistically significant. In addition, no significant differences between the two groups of cats in body weight change were observed.

The choice of the two different regions in which the study was performed, ensured assessment of the product efficacy in different climatic and geographic conditions and with a high environmental infestation pressure, in compliance with the European guidelines.

The sub-optimal comparison between an orally administered product (Credelio™) against a topically applied treatment (Frontline Combo® Spot-on) was driven by the lack of availability of an oral product for cats, which was active against fleas and ticks. The study described in this publication was designed to evaluate efficacy against fleas only. The geographical regions in which the study was conducted were known to have a high prevalence of ticks. Although oral products with efficacy against fleas on cats were available, the sponsor chose not to use those since to have used these products would have exposed the cats to the risks of vector-borne disease transmission from infected ticks. Lotilaner tablets had proven to be efficacious against the main European cat tick (*Ixodes ricinus*) in three pivotal laboratory studies [7] while its efficacy against all ticks of relevance in Europe (*I. ricinus*, *I. hexagonus*, *Dermacentor reticulatus* and *Rhipicephalus sanguineus*), was demonstrated in a large field study performed in three different European countries [16].

Since a topical isoxazoline for cats was not available at the time the study was performed, the applicant decided to choose one of the most commonly used cat parasiticides.

The choice of the comparator product dictated the minimum body weight of the cats for inclusion (1 kg). In the pivotal target animal safety studies, lotilaner was shown to be safe for cats as light as 0.5 kg [9] but since the control product label indicated a higher minimum body weight, in order to maintain the blinding and prevent the introduction of a bias, the minimum body weight of 1 kg at inclusion was selected.

Flea counts and analysis of the demographics and related variables showed that the Credelio™ and Frontline Combo® Spot-on populations were homogeneous at baseline, with the exception of the cat breeds, with a higher percentage of cats of European breed in the Credelio™ group. This was considered of no relevance since the breed *per se* has no impact on the performance of an ectoparasiticide product. The only related variable potentially confounding study results might have been a higher number of cats with long hair in one of the groups, but the comparison of hair length showed that the two treatment groups were not different for this variable, at baseline.

The evaluation of the efficacy against fleas was performed without consideration of the flea species, since *Ctenocephalides felis* is recognised to be the most prevalent species in cats in Europe [17]. For the other relevant European flea species (*Ctenocephalides canis*), a previous *in vitro* study, in which the susceptibilities of European strains of *C. felis* and of *C. canis* to lotilaner were compared in a contact test (unpublished data), had demonstrated an equivalent or higher susceptibility of *C. canis* when compared to C. *felis*. The efficacy of lotilaner against *C. canis* was confirmed in a dose confirmation laboratory study and in a European field study in dogs (unpublished data and [18], respectively). Both studies were pivotal, well controlled, randomized, blinded and performed in compliance with GCP (good clinical practice) standards.

Since there were only ten cats in the lotilaner group and six in the fipronil/(S)-methoprene group showing signs of FAD at baseline, the study has limited power for the non-parametric comparison to baseline in the latter, for the evaluation of the improvement in the clinical signs of FAD. A similar consideration is valid for the non-parametric comparison to baseline in the lotilaner group, with a maximum of five cats showing each sign at baseline, except for pruritus, crusts, and total FAD score, with nine to ten animals affected at baseline. Still, from the analysis within the lotilaner group only, it can be concluded that FAD signs improved substantially over the course of the study.

Administration compliance was 100% in the Credelio™ group, showing that the tablets were easy for pet owners to administer and well accepted by the cats.

## Conclusions

Lotilaner chewable tablets for cats (Credelio™) at the recommended minimum dose rate of 6 mg/kg body weight as a single oral administration in fed state, were shown to be efficacious and safe when administered in the field to client-owned cats. Lotilaner was non-inferior to the approved positive control (Frontline Combo® Spot-on Cat, fipronil/(S)-methoprene) in the treatment of natural flea infestations for 28 ± 2 days on cats presented as veterinary patients in France and Spain. Moreover, Credelio™ was superior to Frontline Combo® Spot-on on both assessment days (14, 28) and for the entire study period (*P* < 0.0001). Analysis of clinical signs of FAD showed that animals treated with lotilaner had significantly lower levels of pruritus, crusts and the total FAD score compared with Frontline Combo Spot-on Cat for the entire study duration. Both products were well tolerated.

## Additional file

Additional file 1: French translation of the Abstract. (PDF 18 kb)

## Abbreviations

AE: adverse event
ANCOVA: analysis of covariance
ANOVA: analysis of variance
CI: confidence interval
FAD: flea allergy dermatitis
GCP: good clinical practice
ITT: intent-to-treat
PP: per protocol
SAE: serious adverse event
SD: standard deviation
VeDDRA: Veterinary Dictionary for Drug Regulatory Activities
