# Supplementary material for: A randomized, controlled field study to assess the efficacy and safety of lotilaner (Credelio™) in controlling fleas in client-owned cats in Europe
Source: Parasit Vectors. 2018 Jul 13;11:410. doi: 10.1186/s13071-018-2971-9 (PMC6044040; doi:10.1186/s13071-018-2971-9)
Supplement: Supplementary file 1 — French translation of the Abstract. (PDF 18 kb) [file 13071_2018_2971_MOESM1_ESM.pdf]

Étude de terrain contrôlée et randomisée visant à évaluer l'efficacité et l'innocuité du lotilaner (Credelio™) pour le contrôle des puces sur des chats de compagnie en Europe

Daniela Cavalleri[1*], Martin Murphy[1], Wolfgang Seewald[1] et Steve Nanchen[1]

[1]Elanco Animal Health, Mattenstrasse 24a, CH-4058, Bâle, Suisse.

*Correspondance : cavalleri_daniela_a@elanco.com

Adresses électroniques :
Daniela Cavalleri, cavalleri_daniela_a@elanco.com
Martin Murphy, murphy_martin_gerard@elanco.com
Wolfgang Seewald, seewald_wolfgang@elanco.com
Steve Nanchen, nanchen_steve@elanco.com

Résumé

Contexte : Le lotilaner est une nouvelle isoxazoline développée comme antiparasitaire externe à administrer par voie orale chez le chien et le chat. De nombreuses études de laboratoire chez le chat ont démontré son innocuité, sa rapidité d'action et la persistance de son activité antipuces et antitiques pendant au moins un mois après administration.
Cette étude avait pour but de démontrer l'efficacité et l'innocuité de comprimés aromatisés à croquer pour chats au lotilaner (Credelio™, Elanco) dans le contrôle des puces en conditions de terrain en Europe.
Méthodes : Dix-sept cabinets vétérinaires situés dans des régions à forte prévalence de puces, en France et en Espagne, ont participé à cette étude. Les foyers sélectionnés, ne comptant pas plus de trois chats et deux chiens, ont été répartis aléatoirement selon un rapport 2/1 entre les deux groupes de traitement, à savoir lotilaner (dose minimale de 6 mg/kg) ou fipronil/(S)-méthoprène topique (Frontline Combo® Spot-on Cats, Merial, administré conformément à la notice). Pour chaque foyer, l'efficacité contre les puces ainsi que les signes de dermatite allergique aux piqûres de puces (DAPP) ont été évalués sur un chat principal (porteur d'au moins cinq puces à J0) ; l'innocuité a quant à elle été évaluée sur

l'ensemble des chats. Les groupes lotilaner et fipronil/(S)-méthoprène comptaient, respectivement, 121 et 61 foyers. Les traitements ont été administrés par les propriétaires des chats à J0. Un comptage des puces et une évaluation de la DAPP ont été réalisés à J0, J14 et J28. L'efficacité était déterminée par le calcul du pourcentage moyen (moyenne géométrique) de diminution du nombre de puces vivantes par rapport au comptage réalisé en début d'étude, préalablement au traitement.

Résultats : L'efficacité du lotilaner a respectivement atteint 97,2 %, et 98,1 % à J14 et J28. Pour ces mêmes jours, l'efficacité du fipronil/(S)-méthoprène était de 48,3 et 46,4 %. Le lotilaner s'est révélé supérieur au fipronil/(S)-méthoprène lors de chacune des évaluations réalisées après J0, ainsi que sur toute la durée de l'étude ($P < 0{,}0001$). Chaque évaluation post-administration a permis de constater qu'au moins 81 % des chats traités par lotilaner ne présentaient pas de puces, contre 25 % dans le groupe fipronil/(S)-méthoprène. Le lotilaner a amélioré ou éliminé les signes cliniques de DAPP, y compris le prurit. Les deux produits ont été bien tolérés.

Conclusions : En Europe et en conditions réelles, les comprimés aromatisés à croquer pour chats au lotilaner ont présenté une efficacité contre les puces supérieure à 97 % ; les signes cliniques de DAPP ont été améliorés ou éliminés. Les comprimés de lotilaner se sont avérés sans danger et ont permis un meilleur contrôle des puces que le fipronil/(S)-méthoprène.

Mots clés : lotilaner, Credelio, puces, fipronil/(S)-méthoprène, Frontline, terrain, chat, efficacité, innocuité, Europe
